# Supplementary material for: Power and influence in world-level sport coaching: A Foucauldian and Raven-informed interpretive vignette study in underwater rugby
Source: PLoS One. 2026 Mar 31;21(3):e0345874. doi: 10.1371/journal.pone.0345874 (PMC13037982; doi:10.1371/journal.pone.0345874)
Supplement: S1 Table — Word file reporting the selected first-line response option for each vignette scenario by each participating coach. (DOCX) [file pone.0345874.s002.docx]

# S1 Table. Scenario by scenario closed choice selections (descriptive).

Note. Each coach completed 10 vignettes. Cells indicate the power base associated with the selected option (French–Raven taxonomy). Counts are descriptive only and are provided to support traceability of Table 3 and the interpretive themes; no statistical inference is intended.

| *Scenario* | *Scenario focus* | *Coach 1* | *Coach 2* | *Coach 3* | *Referent (n)* | *Expert (n)* |
| --- | --- | --- | --- | --- | --- | --- |
| 1 | Authority challenge: player questions decisions during key match | Referent | Referent | Expert | 2 | 1 |
| 2 | Demoralisation post-loss: team demotivated after consecutive defeats | Referent | Expert | Referent | 2 | 1 |
| 3 | Dyadic conflict: two players in constant conflict | Referent | Expert | Expert | 1 | 2 |
| 4 | Communicating new strategy before an important match | Referent | Expert | Expert | 1 | 2 |
| 5 | Underperforming key player | Referent | Expert | Referent | 2 | 1 |
| 6 | Ensuring players understand a new strategy | Referent | Expert | Expert | 1 | 2 |
| 7 | Mid-game disengagement while losing | Referent | Referent | Referent | 3 | 0 |
| 8 | Complacency while leading comfortably | Expert | Referent | Referent | 2 | 1 |
| 9 | Frustration after opponent equalises late | Expert | Referent | Referent | 2 | 1 |
| 10 | Integrating newcomers; veterans uncomfortable | Referent | Referent | Referent | 3 | 0 |
