# Supplementary material for: Power and influence in world-level sport coaching: A Foucauldian and Raven-informed interpretive vignette study in underwater rugby
Source: PLoS One. 2026 Mar 31;21(3):e0345874. doi: 10.1371/journal.pone.0345874 (PMC13037982; doi:10.1371/journal.pone.0345874)
Supplement: S1 Appendix — Word file containing the full vignette questionnaire, including the 10 scenarios and the response-option structure used for data generation. (DOCX) [file pone.0345874.s003.docx]

# S2 Appendix. Vignette questionnaire instrument (scenario prompts; English translation and Spanish original).

This appendix provides the scenario prompts used in the online vignette questionnaire. Participants selected the option that best matched their likely action and then provided a brief open-text justification. To support methodological transparency while protecting confidentiality, the publicly shared version includes the scenario prompts and the decision task structure.

Instructions to participants (English translation):

- You will read 10 recurrent coaching situations. For each situation, select the response that best reflects what you would most likely do in that moment. After selecting, briefly explain why you would respond that way. There are no right or wrong answers; we are interested in your practical reasoning.

Instrucciones a participantes (español):

- Leerá 10 situaciones recurrentes de entrenamiento/competencia. Para cada situación, seleccione la respuesta que mejor refleje lo que probablemente haría en ese momento. Después de seleccionar, explique brevemente por qué respondería de esa manera. No hay respuestas correctas o incorrectas; nos interesa su razonamiento práctico.

Scenario prompts / Situaciones:

## Scenario 1 / Situación 1

Spanish (original):

- Imagina que te encuentras con un jugador que pone en duda tus decisiones durante un partido clave. ¿Cómo respondes?

English (translation):

- Imagine a player challenges your decisions during a key match. How do you respond?

## Scenario 2 / Situación 2

Spanish (original):

- El equipo está desmotivado después de varias derrotas consecutivas. ¿Cómo abordas la situación desde el momento post-competencia?

English (translation):

- The team is demotivated after several consecutive defeats. How do you address the situation immediately post-competition?

## Scenario 3 / Situación 3

Spanish (original):

- Dos jugadores están en constante conflicto dentro del equipo. ¿Cómo actúas para solucionarlo?

English (translation):

- Two players are in constant conflict within the team. How do you act to resolve it?

## Scenario 4 / Situación 4

Spanish (original):

- Necesitas comunicar un cambio en la estrategia del equipo antes de un partido importante. ¿Cómo lo haces?

English (translation):

- You need to communicate a change in team strategy before an important match. How do you do it?

## Scenario 5 / Situación 5

Spanish (original):

- Uno de los jugadores clave está rindiendo por debajo de lo esperado. ¿Cómo manejas la situación?

English (translation):

- A key player is performing below expectations. How do you manage the situation?

## Scenario 6 / Situación 6

Spanish (original):

- Tienes que asegurarte de que todos los jugadores entiendan bien una nueva estrategia. ¿Qué enfoque usas?

English (translation):

- You need to ensure all players clearly understand a new strategy. What approach do you use?

## Scenario 7 / Situación 7

Spanish (original):

- El equipo está perdiendo el partido y los jugadores parecen desmotivados. ¿Qué haces para revertir la situación?

English (translation):

- The team is losing and players appear demotivated. What do you do to turn the situation around?

## Scenario 8 / Situación 8

Spanish (original):

- El equipo va ganando cómodamente, pero aún quedan varios minutos y notas que algunos jugadores están bajando su rendimiento. ¿Cómo gestionas la situación?

English (translation):

- The team is winning comfortably but there are several minutes left and some players are dropping their performance. How do you manage the situation?

## Scenario 9 / Situación 9

Spanish (original):

- El equipo ha estado en ventaja durante la mayor parte del partido, pero el rival acaba de empatar. Los jugadores parecen frustrados y algunos comienzan a perder la calma. ¿Cómo manejas la situación?

English (translation):

- Your team has led most of the match but the opponent has just equalised. Players look frustrated and some are losing composure. How do you handle the situation?

## Scenario 10 / Situación 10

Spanish (original):

- Han llegado varios jugadores nuevos al equipo y algunos de los veteranos parecen estar incómodos con los cambios. ¿Cómo gestionas la integración de los nuevos jugadores?

English (translation):

- Several new players have joined and some veterans appear uncomfortable with the changes. How do you manage integration of newcomers?

## Decision task structure (response option families used for closed-choice tallies).

For each vignette, response options were designed to represent distinct influence logics aligned with French and Raven’s power bases (including informational power). Participants were not shown power-base labels. The following generic option families describe the logic underpinning the closed-choice categories used in Table 3 and S1 Dataset.

- Referent power: prioritise relational connection, reassurance, and identification (trust-building) to realign the group.
- Expert power: draw on technical/tactical expertise via diagnosis, demonstration, and concrete performance cues.
- Informational power: provide brief reason-giving (why/what evidence) to persuade without invoking role authority.
- Legitimate power: invoke role- or norm-based authority to set boundaries/standards or decide unilaterally when required.
- Reward power: offer praise, recognition, incentives, or reinforcement contingent on behaviours/effort.
- Coercive power: threaten or apply sanctions/penalties to deter undesirable behaviour.
