# Supplementary material for: Power and influence in world-level sport coaching: A Foucauldian and Raven-informed interpretive vignette study in underwater rugby
Source: PLoS One. 2026 Mar 31;21(3):e0345874. doi: 10.1371/journal.pone.0345874 (PMC13037982; doi:10.1371/journal.pone.0345874)
Supplement: S1 Analytic Codebook — Word file containing the analytic code structure, code descriptions, and theme-development framework used during qualitative analysis. (DOCX) [file pone.0345874.s004.docx]

# S3 Appendix. Analytic codebook (reflexive thematic analysis of written rationales)

This codebook documents the thematic structure used to organise and interpret the open-text rationales accompanying coaches' closed-choice selections. It is designed for transparency and auditability rather than as a fixed, deductive scheme.

| *Theme / Code* | *Operational definition* | *Include when* | *Exclude when* | *Linked power base(s)* | *Indicative signals (examples)* |
| --- | --- | --- | --- | --- | --- |
| Theme 1: Embodied trust under aquatic risk (Referent power) | Influence enacted through identification, relational credibility, shared rhythm, and presence that stabilise behaviour under risk and time pressure. | Mentions of trust, respect, shared history, exemplarity, calm presence, relational repair, collective identity, or micro-affirmations that secure buy-in. | Purely technical instruction without relational framing; purely rule-based compliance without relational grounding. | Referent (often enabling Expert/Informational) | “I stay close / model calm”; “we reconnect rhythm”; “they follow because they trust the process”. |
| Subcode 1.1: Tempo and emotional re-stabilisation | Rapid relational actions to restore composure, tempo, or confidence after disruption (e.g., conceding, conflict, hesitation). | Brief grounding cues, reassurance, collective reset routines, acknowledgement of emotion, re-focusing under breath-hold constraints. | Extended motivational speeches; generic encouragement without link to situational regulation. | Referent (with occasional Informational) | Short affirmations; “reset”; “breathe and re-enter”; “trust the next action”. |
| Subcode 1.2: Safety-anchored relational governance | Relational influence that is explicitly tied to safety norms (breath management, contact boundaries, risk awareness). | References to safety protocols framed as shared responsibility and mutual care. | Punitive enforcement without relational rationale. | Referent + Bounded Legitimate | “We protect each other”; “safety first, then tactics”. |
| Theme 2: Expertise-as-demonstration (Expert / Informational reasoning) | Influence achieved by credible diagnosis and low-bandwidth teaching, typically through demonstration, modelling, and brief reason-giving that fits the aquatic constraint. | Mentions of showing, modelling, correcting technique, giving concise cues, tactical re-framing with brief “why” statements. | Long explanations not feasible in the setting; purely relational support without instructional content. | Expert (often embedding Informational) | “I demonstrate”; “one key cue”; “show the angle / timing”; “brief why + do”. |
| Subcode 2.1: Micro-brief scripts and signal systems | Pre-agreed short scripts/signals enabling rapid instruction under low-verbal conditions. | References to hand signs, touch cues, pre-breath briefing, single-word commands, standardised gesture sets. | Unstructured talk; improvisational speech without prior shared meaning. | Expert + Informational | “one word”; “signal”; “touch cue”; “pre-breath plan”. |
| Theme 3: Calibrating authority (Selective, bounded recourse to formal power) | Explicit acknowledgement that legitimate/reward/coercive influence is used sparingly, justified by safety, standards, or time-critical coordination. | Mentions of setting rules, enforcing standards, making final calls, applying consequences framed as procedural and transparent. | Coercion as primary mode; threats or punishment without safety/standard rationale. | Legitimate (bounded), occasionally Reward/Coercive (rare, safety-critical) | “non-negotiable standard”; “I step in”; “clear boundary”; “procedurally explained consequence”. |
| Subcode 3.1: Safety-critical override | Coach overrides autonomy when immediate safety is at stake (breath, contact, risky behaviours). | Direct commands justified by risk; immediate stop/restart protocols. | Routine technical correction; relational persuasion. | Legitimate (bounded) | “Stop now”; “reset due to safety”; “rule is non-negotiable”. |
