# Supplementary material for: Power and influence in world-level sport coaching: A Foucauldian and Raven-informed interpretive vignette study in underwater rugby
Source: PLoS One. 2026 Mar 31;21(3):e0345874. doi: 10.1371/journal.pone.0345874 (PMC13037982; doi:10.1371/journal.pone.0345874)
Supplement: Attachment — Submitted filename: Review 1.docx [file pone.0345874.s005.docx]

**Power and influence in world-level sport coaching: A Foucauldian and Raven-Informed phenomenological study**

1. Introduction

Key Weaknesses & Needed Improvements

1. Over-theorisation at the expense of problem clarity
 The introduction is theoretically dense, integrating Foucault, French and Raven, Bourdieu, Gramsci, and Arendt in rapid succession. While intellectually ambitious, this breadth dilutes conceptual focus. The manuscript does not sufficiently justify why these frameworks must be combined, nor how tensions between them are resolved. The result is a sophisticated but diffuse theoretical backdrop rather than a sharply articulated research problem.

2. Limited articulation of empirical gap
 The authors assert that aquatic, low-verbal environments demand different forms of influence, yet do not adequately demonstrate what is missing in existing empirical coaching research. The gap is framed more as a theoretical opportunity than as a clearly evidenced absence in the literature. A more explicit contrast with existing elite coaching studies, especially those already using power or leadership frameworks, would strengthen justification.

3. Aim and contribution are reiterated rather than sharpened
 The study aim is restated in multiple places with similar wording, but without increasing specificity. What exactly is new, a new mechanism, a new typology refinement, or a context-specific instantiation, remains underdeveloped. The introduction would benefit from explicit research questions or propositions to anchor the analysis.

2. Methodology

Design and Philosophical Stance

4. Phenomenological claims exceed the data generated
 Although framed as existential-hermeneutic phenomenology, data were collected via an online vignette questionnaire rather than interviews or embodied observation. This creates a methodological misalignment: phenomenology prioritises lived experience, yet the study relies on hypothetical responses to predefined scenarios. The authors should either (a) moderate phenomenological claims or (b) justify more convincingly how vignette responses constitute lived experience.

5. Vignette design risks theoretical priming
 The scenarios were pre-mapped to French and Raven’s power bases at design time. Despite claims that these were “sensitising concepts,” this structure risks confirmatory bias, subtly steering participants toward theoretically expected responses. This weakens claims of inductive analysis and should be acknowledged more critically.

Participants

6. Extremely small and homogeneous sample
 The sample consists of only three male coaches, all world-champion level. While information power is cited as justification, the manuscript nevertheless over-extends interpretive claims relative to sample size. Gender homogeneity, cultural specificity, and elite exceptionalism are insufficiently problematised. Claims about “coaching practice” should be more explicitly bounded to this niche population.

Data Collection and Analysis

7. Forced single-choice selections oversimplify influence processes
 Each vignette required a single power-base selection, which artificially constrains co-activation, sequencing, and hybridity, ironically central claims of the paper. This design choice directly contributes to the absence of informational, legitimate, reward, and coercive power in the tallies, undermining the robustness of descriptive findings.

8. Reflexive thematic analysis lacks analytic depth in places
 While RTA procedures are correctly cited, theme development remains largely descriptive and confirmatory. The themes mirror the theoretical framing closely, raising concerns that analysis is theory-led rather than data-led. There is limited engagement with ambiguity, contradiction, or deviant cases.

3. Results

Quantitative (Descriptive) Component

9. Frequency tables add limited analytic value
 With n = 3, the presentation of frequencies, percentages, and visual heatmaps risks false precision. Although the authors caution against inference, the visual emphasis may mislead readers. The manuscript would be stronger if descriptive tallies were clearly subordinated to qualitative interpretation, or omitted altogether.

Qualitative Themes

10. Themes insufficiently differentiated
 The three themes, referent trust, expert demonstration, and calibrated authority, are conceptually overlapping. Distinctions between referent and expert power blur in practice, yet the analysis does not sufficiently interrogate this ambiguity. More analytic tension would strengthen credibility.

11. Lack of counter-narratives or resistance
 All accounts portray ethically refined, autonomy-supportive coaching. The absence of conflict, failure, or problematic influence practices suggests social desirability bias, which is acknowledged only briefly. Stronger reflexive interrogation is needed here.

4. Discussion

Theoretical Integration

12. The discussion reiterates rather than extends theory
 While theoretically rich, the discussion largely reaffirms existing Foucauldian and leadership concepts instead of advancing them. The claim that informational power is “subsumed” within expert power is plausible but insufficiently theorised, does this suggest a contextual collapse of power bases, or a limitation of the taxonomy?

13. Limited engagement with alternative explanations
 Other plausible explanations, elite coach professionalisation, cultural norms in underwater rugby, or shared pedagogical training, are mentioned but not analytically explored. This weakens causal plausibility and theoretical contribution.

5. Practical Implications

14. Implications are generic relative to claims of contextual specificity
 Despite strong claims about underwater, low-verbal environments, recommendations for coach education (e.g., trust-building, concise cues, selective authority) are broad and already well-established in coaching literature. More concrete, discipline-specific pedagogical strategies would enhance applied value.

6. Limitations and Future Directions

15. Limitations acknowledged but under-theorised
 Although methodological constraints are clearly listed, the implications of these limitations for knowledge claims are not fully unpacked. For instance, how might vignette-based self-report systematically differ from in-situ behaviour under aquatic risk?
