# Supplementary material for: Power and influence in world-level sport coaching: A Foucauldian and Raven-informed interpretive vignette study in underwater rugby
Source: PLoS One. 2026 Mar 31;21(3):e0345874. doi: 10.1371/journal.pone.0345874 (PMC13037982; doi:10.1371/journal.pone.0345874)
Supplement: Attachment — Submitted filename: Response_to_Reviewers.docx [file pone.0345874.s008.docx]

***Response to Editor and Reviewers (Revised Submission)***

Manuscript title: Power and influence in world-level sport coaching: A Foucauldian and Raven-informed interpretive vignette study in underwater rugby

Corresponding author: Samuel José Gaviria-Alzate

Dear Ms Scaramanga and Editors,

Thank you for the opportunity to revise our manuscript and for the detailed feedback from the staff editor and Reviewer #1. We have made substantive revisions to improve problem clarity, align the methodological framing with the vignette-based design, sharpen the contribution, and strengthen transparency around the descriptive component and data availability.

For ease of review, all changes in the revised manuscript are marked with yellow highlighting and underlining.

A. Staff editor / journal requirements

1. PLOS ONE style requirements and file naming

We checked the manuscript against the PLOS ONE formatting requirements and will follow the journal’s file naming conventions at upload. No content-related changes were required beyond routine formatting consistency.

2. Ethics statement only in the Methods section

We ensured the ethics approval/consent statement appears only within the Methods section and removed any redundant ethics wording elsewhere.

3-4. Data availability and open-data policy (including ‘available on acceptance’)

We revised the Data Availability statement to provide the de-identified closed-choice data underlying Table 3 as Supporting Information (S1 Dataset, CSV) and to provide the vignette prompts/instrument structure (S2 Appendix), analytic codebook (S3 Appendix), and reflexive memo (S4 Appendix). Given the small, readily identifiable elite population, we explain why full open-text rationales cannot be publicly shared without increasing re-identification risk, and we provide an access pathway for qualified researchers to request a further-redacted version under an appropriate data-sharing agreement.

5. Authorship list includes Wilder Geovanny Valencia-Sánchez

We updated the author list to include Wilder Geovanny Valencia-Sánchez.

6. Abstract contains no citations; citations remain in ascending numerical order

We confirmed that the abstract contains no citations. Citations are confined to the body text and retain ascending numerical order on first mention.

7. Include a caption for Figure 1

We included a caption for Figure 1 and updated Figure 1 to a conceptual model aligned with the thematic findings.

8. Reviewer-suggested citations

We evaluated the reviewer’s suggestions and incorporated additional citations only where they directly strengthened the empirical gap and interpretation; we did not add citations that were not materially relevant to the argument.

***B. Response to Reviewer #1 (Jet Clayton Longakit)***

We reproduce each comment in condensed form and respond point-by-point below. Locations refer to the revised manuscript sections; all edits are highlighted/underlined in yellow.

1. Over-theorisation at the expense of problem clarity

We streamlined the Introduction to prioritise the empirical problem (bandwidth-constrained influence in underwater rugby) and the rationale for the two primary lenses (Foucauldian power/knowledge and French-Raven power bases). Additional theoretical signposts (e.g., Bourdieu/Arendt) are now used sparingly to extend interpretation rather than to frame the problem statement. See Introduction; Discussion (theoretical integration).

2. Limited articulation of empirical gap

We strengthened the empirical gap by more explicitly contrasting the underwater, low-verbal context with mainstream elite coaching studies and by specifying what is under-described in the literature: the mechanisms and sequencing of influence when verbal bandwidth is structurally constrained. See Introduction (problem statement and gap paragraphs).

3. Aim and contribution reiterated rather than sharpened

We tightened the study aim and contribution and presented explicit research questions to anchor the analysis. See end of Introduction (research questions).

4. Phenomenological claims exceed the data generated

We aligned the methodological framing with the actual data source by presenting the study as an interpretive qualitative vignette study with a hermeneutic sensibility, rather than as phenomenology based on lived-experience interviews/observation. We moderated any claims that implied direct access to lived experience and clarified what vignette reasoning can and cannot support. See Methods (Design and philosophical stance) and Limitations.

5. Vignette design risks theoretical priming

We expanded our critical acknowledgement of priming risk from the design-time mapping of options to power bases and clarified how we mitigated this risk: power terminology was not shown to participants; rationales were analysed inductively; and the taxonomy was used as sensitising concepts at interpretation. See Methods (Scenario design) and Limitations.

6. Extremely small and homogeneous sample

We further bounded our claims to this niche, elite sample and strengthened the rationale using information power and context-specific aims. We also foreground the implications of gender/cultural specificity as boundary conditions. See Participants; Limitations and Future Directions.

7. Forced single-choice selections oversimplify influence processes

We clarified that the forced single-choice design compresses co-activation and sequencing and therefore cannot be interpreted as prevalence or ‘effectiveness’. To improve transparency, we (i) retained only descriptive counts (Table 3), (ii) provide scenario-by-scenario selections as S1 Table, and (iii) provide the de-identified closed-choice dataset as S1 Dataset. See Methods (Descriptive depiction); Results; Supporting Information.

8. Reflexive thematic analysis lacks analytic depth in places

We strengthened the analytic narrative by sharpening theme boundaries, adding explicit engagement with ambiguity and plausible alternative readings, and expanding reflexive commentary on social desirability and elite self-presentation. See Results (themes) and Discussion (alternative explanations).

9. Frequency tables/visuals add limited analytic value; risk false precision

We removed the heatmap visual emphasis and retained only a compact frequency table (Table 3) as orientation, explicitly subordinated to the qualitative interpretation. Scenario-level detail is placed in S1 Table, and we reiterate that counts are descriptive only with n=3. See Results (Table 3 note; accompanying text) and Supporting Information.

10. Themes insufficiently differentiated

We revised theme descriptions to make the analytic distinctions explicit (relational anchoring vs. expertise-as-demonstration vs. bounded governance), while also acknowledging co-activation and sequencing as an empirical finding rather than a thematic overlap error. See Results (theme introductions and transitions) and Figure 1 (conceptual model).

11. Lack of counter-narratives or resistance

We expanded the reflexive account of why ‘polished’ narratives may appear in vignette self-report and explicitly cautioned readers against equating absence of conflict in these data with absence of conflict in practice. See Results (reflexive paragraph) and Limitations.

12. Discussion reiterates rather than extends theory; ‘subsumed’ informational power needs clearer theorisation

We elaborated the theoretical contribution by interpreting the pattern as a contextual compression/hybridisation of power bases under low-verbal constraints, rather than as a simple absence of informational influence. See Discussion (theoretical integration; taxonomy refinement).

13. Limited engagement with alternative explanations

We expanded the Discussion to more fully consider alternative explanations (elite coach professionalisation, shared pedagogical training, cultural norms and selection effects) and to clarify how these explanations intersect with the underwater ecology account. See Discussion (Alternative explanations and boundary conditions).

14. Practical implications are generic relative to contextual specificity

We revised the Practical implications to include concrete, underwater-specific pedagogical strategies (e.g., minimal cue lexicons, standardised hand/tactile signals, breath-window ‘micro-scripts’, and safety governance protocols). See Practical implications.

15. Limitations acknowledged but under-theorised

We deepened the limitations by explaining how vignette-based self-report may systematically differ from in-situ behaviour under aquatic risk and by specifying how the forced-choice format may suppress co-activation and temporal sequencing. See Limitations and Future Directions.

***C. Supporting Information files provided with this revision***

S1 Dataset (CSV): De-identified closed-choice selections underlying Table 3.

S1 Table: Scenario-by-scenario closed-choice selections (descriptive).

S2 Appendix: Vignette prompts and decision-task structure (English translation and Spanish original).

S3 Appendix: Analytic codebook.

S4 Appendix: Reflexive memo documenting analytic decisions.

Sincerely,

The authors
