# Supplementary material for: Power and influence in world-level sport coaching: A Foucauldian and Raven-informed interpretive vignette study in underwater rugby
Source: PLoS One. 2026 Mar 31;21(3):e0345874. doi: 10.1371/journal.pone.0345874 (PMC13037982; doi:10.1371/journal.pone.0345874)
Supplement: Attachment — Submitted filename: PLOS ONE MINOR .docx [file pone.0345874.s007.docx]

The revised manuscript represents a substantial and thoughtful improvement over the original submission. The authors have clearly engaged with prior critiques and have meaningfully realigned the study’s framing, methods, and claims. In particular, the shift from phenomenology to an interpretive vignette study, the clarification of analytic limits, and the strengthening of reflexivity significantly enhance methodological coherence and transparency.

That said, while the revision is much stronger, some residual conceptual and evidentiary issues remain, particularly regarding the magnitude of the contribution, the role of the closed-choice data, and the extent to which the findings advance theory beyond careful contextual description.

My overall assessment at this stage would be major revisions now satisfactorily addressed, with minor-to-moderate issues remaining.

Major Strengths of the Revised Version

**1. Improved methodological alignment**

The authors’ decision to reframe the study as an interpretive qualitative vignette study with a hermeneutic sensibility is appropriate and convincing. Claims to lived experience have been moderated, and the manuscript now accurately reflects what vignette-based reasoning can support.

**2. Clearer empirical problem and research questions**

The introduction is notably improved. The problem of bandwidth-constrained influence is now clearly articulated, and the research questions provide a focused analytic anchor. The underwater rugby context is no longer merely illustrative but analytically central.

**3. Reflexivity and limitation handling**

The expanded reflexive sections—particularly regarding social desirability, elite self-presentation, and forced-choice compression—demonstrate good qualitative practice. The authors appropriately bound their claims and explicitly caution against over generalisation.

**4. Data transparency and open science compliance**

The revised data availability strategy is thoughtful and appropriate for a small, identifiable elite sample. Providing closed-choice data, instruments, codebooks, and reflexive memos meaningfully strengthens transparency while respecting ethical constraints.

**Remaining Issues and Areas for Improvement**

1. Theoretical contribution remains incremental rather than decisive

While the authors now clearly argue for a contextual compression or hybridization of power bases under low-verbal constraints, the manuscript still stops short of making a clear theoretical move. It remains somewhat ambiguous whether the contribution is:

a refinement of French and Raven’s taxonomy,

a context-specific illustration of Foucauldian power/knowledge,

or a pedagogical model for elite coaching.

At present, it is somewhat of all three, which risks diluting the central contribution. A more explicit statement clarifying what theory is advanced and how would strengthen the manuscript.

2. Closed-choice data still add limited analytic leverage

Although the authors appropriately downgrade the role of frequencies and explicitly warn against over-interpretation, the closed-choice data continue to function mainly as orientation rather than evidence. Given the small sample and forced-choice format, the manuscript might benefit from either:

further integrating these selections into the qualitative interpretation, or

more clearly justifying why their inclusion meaningfully enhances understanding beyond the rationales alone.

As it stands, Table 3 remains methodologically defensible but analytically modest.

3. Conceptual model (Figure 1) risks reification

The new conceptual model is clear and visually coherent, but it may over-stabilise what is explicitly described as dynamic, situational, and contingent influence work. The authors may wish to further emphasise—visually or textually—that the model is heuristic rather than sequential or prescriptive, to avoid reification.

4. Scope of implications could still be more tightly bounded

The practical implications are far more concrete and context-sensitive than before, which is a major improvement. However, some recommendations (e.g., vignette use in coach education, sequencing awareness) extend beyond underwater rugby. A clearer demarcation between context-specific implications and transferable hypotheses would enhance coherence with the study’s stated boundaries.
