# Supplementary material for: Power and influence in world-level sport coaching: A Foucauldian and Raven-informed interpretive vignette study in underwater rugby
Source: PLoS One. 2026 Mar 31;21(3):e0345874. doi: 10.1371/journal.pone.0345874 (PMC13037982; doi:10.1371/journal.pone.0345874)
Supplement: Attachment — Submitted filename: Response to Reviewers.docx [file pone.0345874.s009.docx]

Dear Academic Editor and Reviewers,

We sincerely thank you for the careful reading of our revised manuscript and for the constructive observations that helped us sharpen the contribution, clarify the methodological logic, and better bound our claims. We have revised the manuscript accordingly. In the marked-up manuscript, the changes introduced in this round are shown in blue.

**Academic Editor / overall remaining issues**

**1. Theoretical contribution remains incremental rather than decisive.**

Response: We agreed that the manuscript needed a more explicit and tighter statement of what kind of theoretical move is being made. We therefore revised the end of the Introduction, the opening of the Discussion, and the Conclusion to clarify that the contribution is not presented as a universal revision of French and Raven’s taxonomy, nor as a general pedagogical model of elite coaching. Instead, we now state more directly that the manuscript advances a context-specific refinement for bandwidth-constrained coaching ecologies. Referent and expert power emerge as the dominant actionable pair, informational influence is often nested within expert guidance, and legitimate authority remains a bounded resource for safety and standards.

**2. Closed-choice data still add limited analytic leverage.**

Response: We appreciated this point and clarified the role of the closed-choice data in three places: in the Methodology subsection 'Descriptive depiction of selections', in the opening paragraph of the Results section presenting Table 3, and in 'Methodological contribution'. We now state more explicitly that the tallies are analytically modest on their own but were retained because they preserve what each coach foregrounded when compelled to prioritise a single first-line action. Read alongside the rationales, they provide an orienting layer that helps identify convergence, qualification, and hidden co-activation.

**3. Conceptual model (Figure 1) risks reification.**

Response: We revised the note to Figure 1 to state explicitly that the model is heuristic rather than sequential, exhaustive, or prescriptive. The revised note now frames the figure as a synthesis of the themes under low-verbal aquatic conditions rather than a fixed process model.

**4. Scope of implications could still be more tightly bounded.**

Response: We tightened the first paragraph of 'Practical implications for coach education' and the closing paragraph of that section. The revised wording now distinguishes between direct implications for underwater rugby and comparable low-verbal, risk-aware environments, and broader relevance framed only as a transferable hypothesis for future comparative work rather than a general prescription.

**Reviewer #2**

**1. The rationale for selecting the theoretical frameworks could be clarified further.**

Response: We revised both the Introduction and 'Design and philosophical stance' to explain more clearly why Foucauldian concepts and French and Raven’s taxonomy were combined. We now state that the frameworks were selected because they operate at complementary analytic scales. Foucauldian concepts help interpret the broader governance and normalisation of conduct, whereas French and Raven’s framework offers a mid-range vocabulary for the interactional influence levers visible in concrete vignette responses.

**2. Using a single-optiona data collection format would be beneficial. The rationale for the sample could be explained more clearly.**

Response: We clarified both points. First, we standardised the manuscript language to reflect that each vignette required a single forced-choice response, and we added a methodological justification explaining that this design was used to compel prioritisation of the action coaches would most likely take first under time-pressured conditions. Second, in 'Participants' we expanded the sampling rationale and clarified that this was a deliberately narrow, information-rich elite sample chosen for analytic adequacy rather than representativeness.

**3. Given the limited number of participants, it may be helpful to address how data saturation was taken into account.**

Response: We addressed this directly in the 'Participants' subsection. We now state that saturation was not pursued as a formal endpoint because the study did not aim at broad code saturation across a heterogeneous field. Instead, adequacy was judged in relation to the narrow research question, the rarity of the population, and the density of elicitation generated by ten scenarios per participant.

**4. Further detail on the scenario-based approach would improve methodological transparency (validity and reliability).**

Response: We expanded 'Scenario design' and the questionnaire description to clarify the instrument logic. The revised text now explains the purpose of the design-time mapping, the rationale for the single-option format, and the fact that the vignette guide functioned as an elicitation device rather than as a psychometric scale. We also clarified that methodological attention was directed to contextual realism, face validity, and discriminability of options through expert review, and that the full vignette wording and response structure are supplied in S2 Appendix.

**5. The analytic process could be described in greater detail, particularly regarding how codes were generated and developed into themes.**

Response: We substantially expanded 'Reflexive thematic analysis (RTA)' to describe the analytic sequence more explicitly. The manuscript now outlines six recursive steps: familiarisation, open coding, memo-writing, clustering of related codes, iterative development and renaming of themes, and a final interpretive pass relating themes back to the descriptive selections and the sensitising frameworks. We also added examples of the kinds of action-oriented codes that were grouped into broader thematic patterns.

**6. Including additional illustrative quotations from participants could help demonstrate how themes were derived from the data.**

Response: We carefully considered this suggestion. Because the written rationales were intentionally brief and the sample is a very small, deductively identifiable elite population, we remained cautious about adding numerous standalone quotations that might add limited interpretive depth while increasing disclosure risk. Instead, we strengthened excerpt-to-theme traceability by clarifying the reporting logic in Methods and by linking each theme more explicitly to the scenario clusters and participant-specific patterns that generated it. We believe this preserves interpretive transparency while remaining proportionate to the nature of the dataset.

**7. Inter-rater reliability was not pursued. It would be helpful to clarify whether the analysis conducted was considered sufficient and how analytic rigor was ensured within this process.**

Response: We revised 'Design and philosophical stance' to state more explicitly that, consistent with reflexive thematic analysis, rigour was not sought through coder agreement. We now clarify that analytic rigour was pursued through reflexivity, transparency, memoing, an audit trail, and structured critical-friend interrogation of theme boundaries and evidential adequacy.

**8. The discussion could further explore why referent power and expert power emerged as the most prominent bases.**

Response: We revised the Discussion to elaborate this point more clearly. The new text explains that referent and expert power are especially actionable under low-verbal, time-compressed, breath-constrained conditions. Trust and identification help secure rapid receptivity, while demonstrated competence carries the actionable adjustment. We also added a sentence clarifying why reward, coercive, and even legitimate strategies may be less functional as first-line responses in this particular ecology.

**9. The connection between the theoretical perspective and the analysis could be articulated more clearly.**

Response: We strengthened this connection in the Introduction, Methodology, and Discussion. The revised text now more explicitly links Foucauldian concepts to the governance of conduct and French and Raven’s taxonomy to the interactional prioritisation visible in the scenarios and then reconnects both levels in the Discussion when interpreting the compressed choreography of trust, demonstration, informational guidance, and bounded authority.

**10. Language editing is recommended to improve clarity.**

Response: We carefully edited the manuscript throughout for clarity, consistency, and precision. This included tightening the framing of the contribution, standardising the description of the single forced-choice design, removing residual wording that implied interviews, and smoothing several passages in the Methods, Results, Discussion, and Conclusion.

We hope that these revisions have addressed the remaining concerns and improved the manuscript’s clarity, methodological coherence, and contribution. We are grateful for the opportunity to revise the paper further.

Sincerely,
Samuel José Gaviria Alzate and co-authors
